# Effects of proactive healthcare on pain, physical and activities of daily living functioning in vulnerable older adults with chronic pain: a pragmatic clinical trial with one- and two-year follow-up

**DOI:** 10.1007/s41999-024-00952-9

**Published:** 2024-03-06

**Authors:** Huan-Ji Dong, Anneli Peolsson, Maria M. Johansson

**Affiliations:** 1 Pain and Rehabilitation Centre, Division of Praevention, Rehabilitation and Community Medicine, Department of Health, Medicine and Caring Sciences, University Hospital, Linköping University, 581 85 Linköping, Sweden; 2 Department of Health, Medicine and Caring Sciences, Unit of Physiotherapy, Linköping University, Linköping, Sweden; 3 Occupational and Environmental Medicine Center, Department of Health, Medicine and Caring Sciences, Unit of Clinical Medicine, Linköping University, Linköping, Sweden; 4 Department of Activity and Health, and Department of Health, Medicine and Caring Sciences, Linköping University, Linköping, Sweden; 5 Department of Acute Internal Medicine and Geriatrics, Linköping University, Linköping, Sweden

**Keywords:** Proactive healthcare, Chronic pain, Activities of daily living (ADL), Physical functioning, Vulnerable, Aging

## Abstract

**Purpose:**

To investigate the changes in pain, physical and activities of daily living (ADL) functioning in vulnerable older adults with chronic pain after proactive primary care intervention.

**Methods:**

This study was embedded in a prospective, pragmatic, matched-control multicenter trial at 19 primary care practices in Sweden, with proactive medical and social care (Intervention Group, IG, *n* = 134) in comparison with usual care (Control Group, CG, *n* = 121). Patients with chronic pain, defined as pain experienced longer than 3 months, were included in this subgroup analysis. Data on pain aspects, physical and ADL functioning were collected in the questionnaires at baseline, one- and two-year follow-up (FU-1 and FU-2). Data on prescribed pain medications was collected by local health authorities.

**Results:**

Mean age was 83.0 ± 4.7 years with almost equal representation of both genders. From baseline until FU-2, there were no significant within-group or between-group changes in pain intensity. Small adjustments of pain medication prescriptions were made in both groups. Compared to FU-1, the functional changes were more measurable at FU-2 as fewer participants had impaired physical functioning in IG (48.4%) in comparison to CG (62.6%, *p* = 0.027, Effect Size *φ* = 0.14). Higher scores of ADL-staircase (more dependent) were found in both groups (*p* < 0.01, Effect Size *r* = 0.24 in CG and *r* = 0.16 in IG).

**Conclusion:**

Vulnerable older adults with chronic pain seemed to remain physical and ADL functioning after proactive primary care intervention, but they may need tailored strategies of pain management to improve therapeutic effects.

*Trial registration*: ClinicalTrials.gov 170608, ID: NCT03180606.

**Supplementary Information:**

The online version contains supplementary material available at 10.1007/s41999-024-00952-9.

## Key summary points

**Aim:**

We investigated the changes in pain, physical and activities of daily living functioning in vulnerable older adults (aged ≥ 75) with chronic pain after proactive primary care intervention.

**Findings:**

At two-year follow-up, we found less deterioration in activities of daily living and fewer participants had impaired physical functioning in comparison to those with usual care. No significant difference was found in pain intensity.

**Message:**

Vulnerable older adults seemed to remain physical and activities of daily living functioning after proactive primary care intervention, but they may need tailored strategies for pain management.

**Supplementary Information:**

The online version contains supplementary material available at 10.1007/s41999-024-00952-9.

## Introduction

The aging population is increasing rapidly around the world due to longer life expectancy, medical development, and improvement of health care service [1, 2]. Long life expectancy, apart from the possibilities of a long active life, leads as well to a higher prevalence of age-related diseases and vulnerability [1]. To provide the possibilities for a long active life, effective and accessible health care service is essential. However, today’s healthcare system in many countries has difficulties to meet this need due to the complexity and heterogeneity of this vulnerable population.

Healthcare is usually organized using a reactive approach, aiming to help with health-related symptoms or disorders when they first occur [3]. The reaction is often fragmented without an overview of the multiple conditions, which does not meet the needs of the older patients. Synthesis of existing research suggests that two main factors: accessibility and continuity in primary care (i.e., physician visits) for older patients, correlate with fewer hospitalizations [4, 5]. Accessibility and continuity may contribute to more proactive interventions, by identifying dysfunctions and providing in-time health service. Proactive interventions also involve the prevention of functional decline and maintaining independence. Thus, proactive healthcare in primary care centers may have an opportunity to meet the great challenges for vulnerable older adults.

Clearly, Sweden as well as many other countries, must face challenges in demography and healthcare and thereby need further development and evaluation of primary care interventions for vulnerable older adults. Based on recent evidence and clinical experience, a pragmatical clinical trial with proactive personalized primary care (vs. usual care) for people 75 years and older in the prevention of future hospitalization was designed and started 2017, followed by a 2-year continued intervention phase [3]. In this large trial, we focused on a subpopulation of patients with chronic pain condition (pain duration ≥ 3 months) due to its high prevalence [6], numerous negative health consequences (e.g. impaired functioning, well-being and ability in daily activities) [7–9], and heavy economic burden for both society and individual [10, 11]. Furthermore, proactive intervention in chronic pain is warranted [9, 12] and has not been investigated before. Hence, we aimed to investigate the changes in pain, physical functioning, and ability in everyday life from the baseline year prior to a team-based intervention (screening and personalized proactive intervention by a physician or a nurse) in primary care centers, to one- and two-year after the intervention. For this subpopulation of patients with chronic pain, we hypothesized that this proactive primary care intervention would result in better functional outcomes and more optimal pain medications than usual care. To achieve optimal medication use, the adjustments of prescribing and deprescribing should be considered [13].

## Methods

### Study population

The detail of patient recruiting, intervention and follow-up procedure was published as a study protocol [3]. We used a recently developed and validated case-finding algorithm (prediction for hospital care) that contains 38 variables identified with multivariable logistic regression to identify vulnerable older persons aged 75 years and older in Southeast Sweden [14]. Vulnerable older adults (*n* = 1600, about 36% of the entire region), based on the calculation from this predictive model (reduced hospitalization by 20% as a primary outcome) and a pilot study of drop-outs estimation, constituted the 11% with the highest risk score of the target population [15]. The vulnerable patients were listed in 19 Primary Care Centers in the county of Östergötland, Sweden. The intervention was provided by nine centers (Intervention Group, IG). Patients in the other ten healthcare centers received care as usual (Control Group, CG) and these centers were selected to match in terms of location (city and countryside), size and socioeconomic distribution. There was no randomization of healthcare centers or at patient levels.

**Fig. 1:**
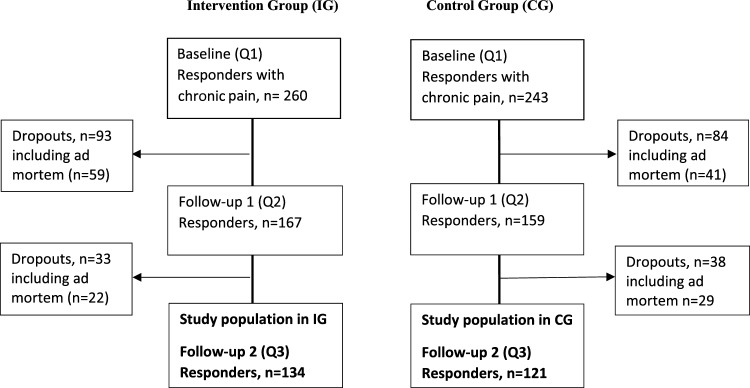
Flowchart study population

After receiving a postal questionnaire and an invitation letter containing an informed consent for participation (*n* = 1487, ad mortem *n* = 113), a total of 853 (57%) responded and filled in the baseline questionnaire (IG = 440 and CG = 413). For this subgroup analysis, we included responders with pain persisting or recurs for more than three months, according to the International Association for the Study of Pain (IASP) classification of chronic pain [16]. Out of those 503 responders who reported chronic pain, over half (*n* = 255) also completed the postal-questionnaires on the latter two occasions: 1-year (FU-1) and 2-year follow-up (FU-2). The proportion of responders in both groups on each occasion was approximately equal (Fig. 1). The study was approved by the regional ethical review board in Linköping (Dnr 2016/347-31). All participants filled in an informed consent prior to participation.

### Intervention

The proactive healthcare, led by patients’ responsible general practitioner (GP), consists of a health assessment using the standardized evaluation form —the Primary Care Assessment Tool for the Elderly (PASTEL) [15], to identify the health-related problems for need of medical treatment, rehabilitation, or prevention. PASTEL is constructed for the project and is based on the holistic approach of Comprehensive Geriatric Assessment (CGA) [14]. PASTEL combines the diagnostic and therapeutic purposes, covering medical, functional, psychological, and social domains [15]. An overview of patients’ medical history including an evaluation of current medication use as well as physical measures were included in the clinical assessment. The GPs and nurses were introduced to the CGA tool and the process of the intervention prior to the study. When needed (based on this CGA and clinical assessment) referrals were done to other professions such as physiotherapist (PT), occupational therapist (OT), dietitian or social caregiver. CGA-related issues and experiences were discussed and shared in the regular seminars every 6 months from the baseline year to the 2nd year follow-up. When referrals were made to OT or PT, individual pragmatic assessments, rehabilitation, and prevention were done as well. For example, this team could provide individually tailored and home-based exercise program after a home visit. The need for assistance technology (e.g., walker, adapted toilet, bath/shower technology) and the extent to which assistance (i.e., transportation service, community assistance, and personal alarm) could also be identified to facilitate everyday life. PASTEL includes suggestions for different interventions but there was no standard procedure when to refer to other professions. No standard intervention except for the CGA assessment within the project was offered. All were made on an individual basis and follow-ups were also done individually by their GPs and nurses within the project. Seventy-four percent (*n* = 475) accepted the invitation to the CGA assessment, followed by 67% (*n* = 345) at FU-1 and 65% (*n* = 334) at FU-2. In the Control Group, the vulnerable patients received routine medical consultations (usual care) from their caregivers in the primary care. The consultations were arranged by GPs to treat medical conditions when a patient sought or got referred to the primary care center by another specialist. There was no interaction with the patients in CG within the study. All participants were invited to complete the postal questionnaires which covered health aspects on up to three occasions: during the first invitation (baseline), at 1-year (FU-1) and 2-year follow-up (FU-2).

### Outcome

This subgroup analysis specifically focused on the changes of pain-related outcomes such as pain intensity, prescription of pain medications, physical functioning, general health, and activities of daily living (ADL). We also made comparisons of socio-demographic factors between IG and CG since sociodemographic disparities may affect these outcomes.

### Measures

Data presented in the current study consists of the baseline data as well as data during 1- and 2-years follow-up from the postal questionnaires and computerized information system of the local health authority, County Council of Östergötland, Sweden. The information on the age and gender of one person were from Statistics Sweden (SCB).

#### Socio-demographic factors

Age (years), gender, marital status (currently married, unmarried, or widows), living situation (living alone, together with partners, or with children), housing (own housing, sheltered accommodation, or nursing home), highest education (less than nine years’ of schooling, nine-years of schooling, secondary school, or university /college) were collected at baseline year.

#### Pain duration

How long have you been suffering from pain? (months). The answer at baseline year was used to identify persons with chronic pain [16].

#### Pain intensity

Pain intensity over the preceding seven days was measured using a visual analog scale (Pain-VAS, 0 = no pain and 100 = worst imaginable pain) [17].

#### Common medications prescribed for pain management

Medications were coded into different groups according to the WHO Anatomical Therapeutical Chemical classification index. Data sources during the study period were obtained from the computerized information system of the County Council of Östergötland. Analgesics were grouped into the following categories: paracetamol (N02BE); non-steroidal anti-inflammatory drugs (NSAID) (M01A); topical agents (M02A); opioid analgesic (N02A); and others including migraine medications (N02C) and antigout preparations (M04A). Tramadol (N02AX02) and Tapentadol (N02AX06) were included in the N02A group. We also include medications that are commonly prescribed in chronic pain treatment, including antiepileptics (N03AX12, N03AX16 and N03AF01), antidepressants (N06AA, N06AB, and N06AX), medications for insomnia (N05CD, N05CF, N05CH, N05CM06, and N05CM02), and muscle relaxants (M03BB, M03MC and M03BX).

#### EQ-VAS

The EQ-5D is a generic instrument that assesses health-related QoL (HRQoL) [18, 19]. The individual’s self-rated general health was measured using a vertical VAS/thermometer ranging from 0 (worst imaginable health status) to 100 (best imaginable health status) [20].

#### RAND-36 physical functioning (RAND-36_PF_)

RAND-36 is one of the generic profile HRQoL and a subscale with 10 items was used to determine physical functioning (Rand-36_PF_) [21]. Higher scores (range: 0–100) indicated better physical functioning. Impaired function refers to a decreased score over time. Good internal consistency (Cronbach’s alpha of 0.92) was demonstrated in the present study.

#### Activities of daily living (ADL)

ADL-staircase is an extended version of Katz´ ADL index [22] that contains 10 activities covering both Basic ADL (BADL) and Instrumental ADL (IADL) [23]. Each activity has a three-graded scale of dependency (0 = independent, 1 = partly dependent and 2 = dependent). A total sum from 0 to 20 is calculated with a higher score indicating greater dependency, therefore impaired function refers to a higher score of ADL over time. Scores of BADL (0–12) including feeding, continence, transfer, toilet use, dressing, bathing and IADL (0–8) including cooking, shopping, cleaning, and transportation were also generated to evaluate the dependency and to evaluate any differences in dependency. Good construct validity has been reported previously [24]. Good internal consistency (Cronbach’s alpha of 0.96) was demonstrated in the present study.

### Statistical analysis

Analyses were performed using the statistical package IBM SPSS Statistics (version 26.0; IBM Inc., New York, USA). All data are reported as mean and standard deviation (SD), median with interquartile range (IQR), or number with percentage based on the distribution of data. Kolmogorov–Smirnov tests as well as histograms were used to determine if data were normally distributed. Chi-square test (or Fisher’s test) and Mann–Whitney U-test were used between the two groups and Wilcoxon signed rank test and Cochran’s Q test for comparison within the groups. When appropriate, effect size (ES) was computed to describe the magnitude of differences between the two groups or within group (IG or CG). For chi-square test (*φ* = √(*x*^2^/*N*) and Wilcoxon signed-rank test (*r* = *z*/√*N*), the* φ or* *r* varies between 0 and 1 (lower than 0.1 = no effect, 0.1–0.29 = “small effect”, 0.3–0.49 = “moderate effect” and 0.5 or higher = “large effect”) [25].

In case that missing data can potentially lead to biased results, we also presented the baseline results for non-responders and dropouts at the follow-up occasions (supplementary material).

## Results

### Sociodemographic backgrounds

Of the 255 participants who completed the measures during the study period, the overall mean age at baseline was 83.0 ± 4.7 years with almost equal representation of both genders. Nearly half of the respondents were couples (married or cohabited) and living together. Only 2% lived in their sheltered accommodation/nursing homes. A minority of this sample had a university or college education (48, 19.3%). No statistical difference was found between IG and CG (Table 1).

**Table 1:** General characteristics of the study population (*n* = 255) with chronic pain at baseline

	Total, *N* = 255	Intervention Group, *n* = 134	Control Group, *n* = 121	*P*-value
Age (years), mean ± SD	83 ± 4.7	83 ± 4.6	83 ± 4.8	0.990
75–84, *n* (%)	164 (64.3)	82 (61.2)	82 (67.8)	0.274
85+, *n* (%)	91 (35.7)	52 (32.2)	39 (32.2)	
Gender, male, *n* (%)	142 (55.7)	76 (56.7)	66 (54.5)	0.727
Marital status	*n* = 253			0.933
Currently married	146 (57.7)	77 (57.5)	69 (58)	
Unmarried or widows	107 (42.2)	57 (42.5)	50 (42)	
Living situation (*n* = 254), *n* (%)	*n* = 254			
Living alone	104 (40.9)	56 (41.8)	48 (40)	0.772
Living with partners/children	150 (59.1)	78 (58.2)	72 (60)	
Housing, *n* (%)				
Own house/apartment	250 (98)	133 (99.3)	117 (96.7)	0.193
Sheltered accommodation/nursing home	5 (2)	1 (0.7)	4 (3.3)	
Education levels, *n* (%)	*n* = 249			
Less than 9 years’ school	95 (38.2)	52 (40)	43 (36.1)	0.617
9-year school	19 (7.6)	12 (9)	7 (5.9)	
Secondary school	87 (34.9)	42 (32.3)	45 (37.8)	
University/college	48 (19.3)	24 (18.5)	24 (20.2)	

*SD* standard deviation

Compared to the dropouts during the study period (*n* = 248), the participants were more likely to be younger (*p* < 0.001), currently married (*p* = 0.003) and living with partners or children (*p* = 0.001) and residing in own housing /apartment (*p* < 0.001) (Supplementary material, Table 1). The proportion of gender and different education levels were similar between the dropouts and participants (*p* > 0.05).

### Pain aspects

**Fig. 2:**
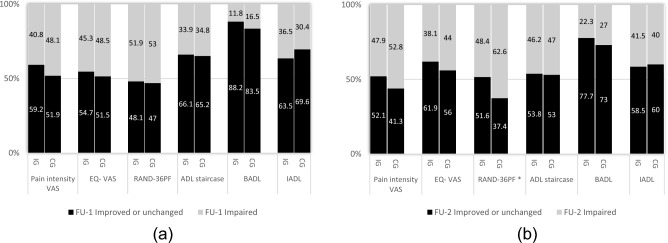
**a** Percent of patients with difference between baseline and 1st year follow-up (FU-1). Intervention group vs control group. **b** Percent of patients with difference between baseline and 2nd year follow-up (FU-2). Intervention group vs control group, *p < 0.05. *VAS* visual analogue score, *RAND-36*_*PF*_ RAND-36 physical functioning, *ADL* activities of daily living, *BADL* basic activities of daily living, *IADL* instrumental activities of daily living

The mean pain duration at baseline was similar in IG (24.7 ± 2.1 months) and CG (25.1 ± 2.3 months, *p* > 0.05). In IG, the median values of pain intensity VAS varied between 34 (IQR 20–61) at FU-1 and 42.5 (IQR 24.7–60) at FU-2, but they did not significantly differ from the median value of 40 (IQR 21–59) at baseline (*p* > 0.05, Table 2). Similarly, participants in CG reported a slight but non-significant impairment (increased VAS) on the three occasions. Moreover, no statistical difference was shown between the two groups on any measured occasion (*p* > 0.05). As shown in Fig. 2a, b, we neither found any statistical significance during the follow-up years regarding the proportions of participants who rated impaired pain (increased VAS) between the two groups.

**Table 2:** Changes of pain intensity, HR-QoL and ADL from baseline to one- and two-year follow-up (FU-1 and FU-2)

	Baseline (A) Median (IQR)	FU-1 (B) Median (IQR)	FU-2 (C) Median (IQR)	*P* value (z) A versus B, A versus C
Intervention Group, *n* = 112–132				
Pain intensity-VAS	40 (21–59)	34 (20–61)	42.5 (24.7–60)	0.24 (*z* = − 1.18), 0.82 (*z* = − 0.23)
EQ-VAS	53 (40–70)	60 (40–70)	60 (40–70)	0.51 (*z* = − 0.66), 0.38 (*z* = − 0.88)
RAND-36_PF_	40 (20–63.7)	35 (20–65)	45 (15–60)	0.18 (*z* = − 1.35), 0.19 (*z* = − 1.30)
ADL-staircase	2.5 (0–6)	3 (0–6)	3 (1–6)	0.64 (*z* = − 0.64),** 0.009 (***z* = − **2.62)**
BADL	0 (0–1)	0 (0–1)	0 (0–1)	0.57 (*z* = − 0.57), 0.13 (*z* = − 1.51)
IADL	2 (0–5)	2 (0–5)	3 (1–5)	0.25 (*z* = − 1.15), **0.01 (***z* = − **2.54)**
Control Group, *n* = 101–118				
Pain intensity-VAS	38 (26–59.7)	42.5 (23–66.7)	48 (24.5–67)	0.52 (*z* = − 0.64), 0.33 (*z* = − 0.98)
EQ-VAS	60 (50–79.5)	60 (40–72.5)	60 (48.5–70)	0.14 (*z* = − 1.49), 0.44 (*z* = − 0.77)
RAND-36_PF_	45 (20–70)	42.5 (20–65)	35 (15–60)	**0.03 (***z* = − **2.16), 0.001 (***z* = − **3.44)**
ADL-staircase	3 (0.7–6)	3 (0–6)	3.5 (1–8)	0.37 (*z* = − 0.90), < **0.001 (***z* = − **3.60)**
BADL	0 (0–2)	0 (0–1)	0 (0–2)	0.70 (*z* = − 0.39), **0.051 (***z* = − **1.95)**
IADL	2 (0–5)	2 (0–5)	3 (1–6)	0.47 (*z* = − 0.72), < **0.001 (***z* = − **3.74)**

Significant values are in bold

*IQR* median with interquartile range, *VAS* visual analogue score, *RAND-36*_*PF*_ RAND-36 physical functioning, *ADL* activities of daily living, *BADL* basic activities of daily living, *IADL* instrumental activities of daily living

**Fig. 3:**
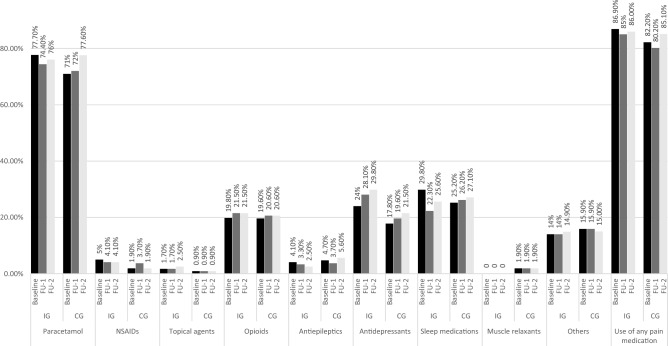
Prevalence of pain medications in intervention and control groups at baseline, one- and two years follow-up (FU-1 and FU-2). *NSAIDs* nonsteroidal anti-inflammatory drugs

Regarding the prescription of pain medications from baseline to FU-2, no statistically significant changes were found in any of the categories between the two groups or within Intervention/Control Group (Fig. 3). Figure 3 lists the common medications for treating chronic pain conditions. Paracetamol was the most common analgesics, prescribed for 77% and 71% patients in IG and CG, respectively. We also observed a stable opioid consumption rate (approximately 20%) and few prescriptions of NSAIDs (≤ 5%). Among the adjuvant analgesics, we observed that antidepressants prescription slightly increased by 5.8% and 3.7% in IG and CG, respectively. In IG, a decline rate of insomnia medication consumption was found at FU-1 by 7.5%. Finally, we noted a tiny increase rate of any pain medication use by 3% in CG at FU-2 whilst it remained almost unchanged over time in IG.

### General health, physical functioning, and ADL

Median values of EQ VAS (53–60-60) and Rand-36_PF_ (40–35-45) had an increasede trend in IG from baseline to FU-2, but there was no statistical significance in the change over time (Table 2). In CG, the significant decrease median score of Rand-36_PF_ was reported at FU-1 (*p* = 0.03) and FU-2 (*p* = 0.001), in comparison to that at baseline (median values at baseline, FU-1 and FU-2: 45–42.5–35). At FU-2, there was a slightly higher proportion of participants in CG with impaired physical functioning than those in IG (Fig. 2b, p = 0.027, *φ* = 0.14). The absolute scores measured on each occasion, however, showed no statistical difference between the two groups (*p* > 0.05).

Participants in both groups rated significantly higher ADL-staircase (more dependent) at FU-2 compared with the scores at baseline (*p* < 0.01, Table 2). The ES of difference in ADL scores was relatively larger in CG (*r* = 0.24) than that in IG (*r* = 0.16). This difference was mainly attributed to the increased scores of IADL at FU-2 with small ES in IG (*r* = 0.16, *p* = 0.01) and CG (*r* = 0.25, *p* < 0.001). Notably, a marginal significant difference (small ES) of BADL at FU-2 compared to that at baseline was found in CG (*r* = 0.13, *p* = 0.051). However, the proportions of participants with unchanged or improved ADL were similar in both groups at FU-1 (Fig. 2a) and FU-2 (Fig. 2b). Regarding the absolute ADL values measured on the three occasions, no statistical difference was found between the two groups (*p* > 0.05).

## Discussion

This study presented the changes in pain, ADL, and HRQoL in vulnerable older adults with chronic pain after the CGA intervention. The effects were somehow more measurable at the 2nd-year follow-up (FU-2). Compared to CG, fewer participants in IG had impaired physical functioning at FU-2, but no significant changes in pain intensity and self-rated general health were found between groups from baseline until FU-2. The small increased degree of ADL-staircase score (more dependent) was found within a group at FU-2 and less deterioration was found in IG. Regarding optimizing pain medication prescriptions, no statistically significant difference was found in the prescription change between IG and CG. This study expands our understanding of the difficulty in clinical practices for vulnerable older adults.

Chronic pain per definition is a health condition for the group in question. Based on the purpose of intervention, proactive healthcare facilitates a holistic perspective where pain management (pharmacological and non-pharmacological) is supposed to be considered. Medication reviews together with a CGA seem to be beneficial to optimize the drug consumptions in IG as we observed a slight adjustment of adjuvants and sleep medications. However, these small changes did not show statistical significance. The GPs in both groups were also strictive with NSAIDs prescription and preferred paracetamol, in accordance with the current recommendation [26]. These findings did not differ between the groups. One explanation might be the medication reviews included in usual case also benefited participants in CG according to the regulation from The Swedish National Board of Health and Welfare that drug utilization reviews should be offered at least once a year to patients 75 years of age or older prescribed at least five drugs [27, 28]. It is well acknowledged that multiple pharmacologic agents might not always be of help [29, 30], non-pharmacological approaches, such as referrals to the rehabilitation team (PT, OT, dieticians, etc.) were supposed to be included in the team-based interventions for pain management. We might not expect a reduction of pain intensity since this outcome was also controversial in other trials with short-term follow-up [31, 32] and functioning improvements were not always associated with reduced pain intensity [33, 34]. We can only speculate about why there was no difference between the groups regarding pain intensity, and there is a need to investigate this further. One possible reason could be that the focus of involved healthcare professionals was on health conditions with well-known diagnosis and there was a lack of knowledge regarding pain and pain management. Knowledge of what physiotherapists and occupational therapists can contribute with in pain rehabilitation may also be inadequate among the doctors and nurses who were in contact with the patients as shown in a few referrals to rehabilitation personals. Another reason might be that this group of patients might have had their pain for a long time and gone through different interventions already. Multimorbidity is extremely common in old age, and medications might be difficult to optimize for reaching pain relief only measured by pain intensity. Additionally, lack of motivation for other therapies than medications might also be an explanation [35].

It is complex to follow the effect of an intervention for a group of vulnerable older patients over time where the natural course due to age, the burden of comorbidity and changes in living situation can be expected to lead to deterioration. These characteristics related to deterioration were reported in other observational studies [36, 37]. In our study population, a reduced deterioration due to intervention can therefore be regarded as positive. For this reason, the results of the current study regarding physical functioning and ADL are to be regarded as both positive and of great importance. This finding matches one of the main goals of proactive healthcare, to delay or prevent deterioration of functional status and ADL ability by helping vulnerable older patients address and manage health problems prior to the time of being reactive (acting when health problems occur) [3].

One strength of this study is that we collected data from a trial using a pragmatic design of the intervention and it allowed us to study the effects of intervention from real-world settings. There were no planned consultations or measurements of the controls in everyday clinical practice, otherwise they could be considered as confounders and mask the true effect [38]. Moreover, PASTEL offered freedom to the GPs and nurses to tailor further assessments and actions to the individual in need. No standard or experimental treatments in this trial were planned in IG. Hence, the pragmatic trial design provided reliable evidence to meet the need for the management and care of vulnerable older people with chronic pain. Another strength is the access to registered data of prescribed medication that allows us to avoid recalled bias, possible error of proxy estimation, or considerable missing data [39].

Some limitations need to be considered. First, the ADL-staircase investigates basic ADL and four IADL activities but does not include more complex activities that might be important to older people. Despite this limitation, it is a worthful and well-known instrument, investigating older vulnerable adults` function. ADL abilities are also affected by a lot of other factors such as environmental and sociopsychological factors [40]. The aspect of self-reported data must be considered as well. Some participants might over or underestimate their functional abilities in cases of cognitive impairment [41]. On the other hand, patient-reported outcomes are important to reflect one’s own belief of the function and ability. In addition, a strength is that the same outcomes were used at follow-ups. Functional assessments together with self-reported measures can provide more information in future studies.

Second, the power calculation was not based on the outcomes of this study, but on another primary outcome of the trial: the hospitalization [3]. The study was designed pragmatically in the recruitment of participants, so the lack of difference between the groups over time may be affected by insufficient power to show equivalence. Many participants (80/134, 59.7%) in IG received more interventions due to the CGA process, but only a few received interventions from physiotherapists (*n* = 16, 20%) and occupational therapists (*n* = 7, 9%) at the follow-up. Since the main study did not focus on pain management per se and the CGA process was on a holistic approach, we do not know if these actions were taken towards pain management and how long the therapeutical interventions lasted. We followed the participants up to two years, which is longer than usual follow-ups for working-age populations in pain rehabilitation (12 months) [42].

Third, this study specifically included all participants who completed the questionnaires by themselves or by proxy. Only a minority of older people with severely impaired cognitive function could participate if they got help from close relatives or caregivers due to reduced autonomy. The non-responders and dropouts could include frailer persons with reduced autonomy than the participants. Finally, we did not classify chronic pain as primary (e.g., fibromyalgia) or secondary pain (e.g., cancer-related pain). A main distinction from studies on younger adults who often suffer from primary pain, we suppose the most chronic pain in older adults are secondary to another previous disorder (i.e. cancer, neuropathic pain, musculoskeletal changes, posttraumatic or postsurgical pain, visceral pain, headache and orofacial pain) [43, 44]. This statement could partly be confirmed by the analysis of pain locations in our previous study on the same population [45]. An identification of the pain etiology (classification) in future clinical research could guide tailored interventions to the target patients. If the pain etiology is difficult to approach, researchers and clinicians may consider multisite pain as a 'geriatric syndrome' to provide holistic management [46].

In conclusion, proactive primary care intervention provided a holistic perspective of health assessment followed by essential medical treatments, rehabilitation and/or prevention for vulnerable older people. For those with chronic pain, more participants in IG than those in CG remained physically functioning and had less deteriorated ADL, but this proactive intervention did not significantly change pain intensity, general health, or functions at FU-1 and FU-2. The small changes enhance our understanding of the difficulty in clinical practices for vulnerable older people. More targeted treatments and pain rehabilitation warrant further consideration to improve therapeutic effects in old age with vulnerability.

### Supplementary Information

Below is the link to the electronic supplementary material.

Supplementary file1 (DOCX 27 KB)
