# Supplementary material for: Effects of proactive healthcare on pain, physical and activities of daily living functioning in vulnerable older adults with chronic pain: a pragmatic clinical trial with one- and two-year follow-up
Source: Eur Geriatr Med. 2024 Mar 6;15(3):709–18. doi: 10.1007/s41999-024-00952-9 (PMC11329693; doi:10.1007/s41999-024-00952-9)
Supplement: Supplementary file 1 — Supplementary file1 (DOCX 27 KB) [file 41999_2024_952_MOESM1_ESM.docx]

**Supplemental material:**

**Table 1 Differences of sociodemographic background between participants and dropouts**

|  | **Participants, N=255** | **Dropouts, n=248** | **Participants vs dropouts, *p*-value** |
| --- | --- | --- | --- |
|  |  |  |  |
| Age (years), mean±SD | 83±4.7 | 85.3±6.0 | <0.001 |
| 75-84, n (%) | 164 (64.3) | 111 (44.8) | <0.001 |
| 85+, n (%) | 91 (35.7) | 137 (55.2) |  |
| Gender, male, n (%) | 142 (55.7) | 231 (51.3) | 0.084 |
|  |  |  |  |
| Marital status | n=253 | n=242 | 0.003 |
| Currently married | 146 (57.7) | 107 (44.2) |  |
| Unmarried or widows | 107 (42.2) | 135 (55.8) |  |
| Living situation (n=254), n (%) |  |  | 0.001 |
| Living alone | 104 (40.9) | 137 (55.9) |  |
| Living with partners or children | 150 (59.1) | 108 (44.1) |  |
| Housing, n (%) |  |  | <0.001 |
| Own house /apartment | 250 (98) | 215 (88.1) |  |
| Sheltered accommodation /nursing home | 5 (2) | 29 (11.9) |  |
| Eduction levels, n (%) | n=249 | n=240 | 0.052 |
| Less than 9 years’ school | 95 (38.2) | 113 (47.1) |  |
| 9-year school | 19 (7.6) | 24 (10) |  |
| Secondary school | 87 (34.9) | 74 (30.8) |  |
| University/college | 48 (19.3) | 29 (12.1) |  |
